# Maternal Talk in Cognitive Development: Relations between Psychological Lexicon, Semantic Development, Empathy, and Temperament

**DOI:** 10.3389/fpsyg.2016.00394

**Published:** 2016-03-22

**Authors:** Dolores Rollo, Francesco Sulla

**Affiliations:** Department of Neuroscience, University of ParmaParma, Italy

**Keywords:** psychological lexicon, shared reading, semantic development, children's temperament, mother's empathy

## Abstract

In this study, we investigated the relationship between mothers' psychological lexicon and children's cognitive and socio-emotive development as assessed through conceptual and semantic understanding tasks, in addition to the traditional tasks of theory of mind. Currently, there is considerable evidence to suggest that the frequency of mothers' mental state words used in mother-child picture-book reading is linked with children's theory of mind skills. Furthermore, mothers' use of cognitive terms is more strongly related to children's theory of mind performances than the mothers' references to other mental states, such as desires or emotions (Rollo and Buttiglieri, 2009). Current literature has established that early maternal input is related to later child mental state understanding; however it has not yet clarified which maternal terms are most useful for the socio-emotional and cognitive development of the child, and which aspect of the cognitive development benefits from the mother-child interaction. The present study addresses this issue and focuses on the relationship between mothers' mental state talk and children's behavior in conceptual and semantic tasks, and in a theory of mind task. In this study fifty pairs consisting of mothers and their 3 to 6-year-old children participated in two sessions: (1) The mothers read a picture book to their children. To assess the maternal psychological lexicon, their narrative was codified according to the categories of mental state references used in literature: perceptual, emotional, volitional, cognitive, moral, and communicative. (2) After a few days, the conceptual and semantic skills of the children (tasks of contextualization and classification, memory, and definition of words) and their psychological lexicon were assessed. The results suggest close links between the frequency and variety of mothers' mental state words and some semantic and conceptual skills of children.

## Introduction

### Mother-child talk about inner states and the development of theory of mind

There is an abundance of studies showing that language, and in particular, the use of it in social interactions, is at the core of the processes involved in mind understanding. “Primitive access to the social-cultural world is available through participation in its routines, but access to the ways in which the world semiotically structures concepts, ideas, frames, and theories is available only through language. Thus the cultural system reflected in adult ideas about others' mental states becomes more visible to children as they participate as language users in culturally constituted activities (games, routines, work, commerce, storytelling)” (Nelson, 1996, p. 312). Language constitutes systems of symbols conventionally used in constructions that convey meaning between people (Aitchison, 2012). People use, and children learn to use, varying systems for talking together in different settings.

Children can learn to think about their experience and to interpret it from the conversations with their mothers, and certainly, narratives about the past and the future in the children's experience incorporate talk about mental states. Parents tend to treat the children as social partners and conversationalists almost from birth, and children respond with attentive looks, gurgles, smiles. This practice is important to the children's entering into meaningful communicative exchanges (Astington and Jenkins, 1999; Nelson, 2005). A current cognitive development area of great current interest is children's theory of mind while the semantic domain corresponding to the purported theory is that of internal state terms. In studying the linguistic correlates of the theory of mind, particular attention has been devoted to psychological lexicon or mental-state language, a type of talk that several studies (e.g., Bretherton and Beeghly, 1982; Bartsch and Wellman, 1995; Camaioni et al., 1998) have classified in the following categories: physiological (e.g., to be hungry, to be thirsty, to be sleepy), perceptual (e.g., to hear, to see, to look, to observe, to recognize, to be cold, to be hot, to feel ill), emotional (e.g., to love, to enjoy, to be afraid, to be sorry), volitional (e.g., to want), cognitive (e.g., to know, to understand, to remember, to think), communicative (e.g., to say, to tell, to call), and moral (e.g., duty = to be obliged to do, power = to have the permission to be good to to be bad).

Children's language is important in assessing the development of their mental states (Rollo, 2007). The development of mental state words has been investigated by a number of researchers (Bretherton and Beeghly, 1982; Wellman and Bartsch, 1988; Wellman, 1991; Bartsch and Wellman, 1995) for clues to children's understanding of the mind, with the assumption that the use of such words (especially *know* and *think*) refers to internal states reflecting an organized theory of those states (Nelson and Kessler Shaw, 2002; Meins et al., 2006; Lecce et al., 2010).

Plenty of research conducted on children's development of social cognition has examined the relations between mother's mental state language produced during parent-child book reading, and children's psychological lexicon (Dunn et al., 1987; Dunn, 2002; Ruffman et al., 2002; Pons et al., 2003; Adrian et al., 2005; de Rosnay et al., 2004; de Rosnay and Hughes, 2006; Taumoepeau and Ruffman, 2006, 2008; Slaughter et al., 2007; Hughes, 2011; Ziv et al., 2013). Also examined was the connection between the children's mental state language and their performance on tests of theory of mind understanding (e.g., false-belief tasks, Wimmer and Perner, 1983). These studies have shown that mother-child conversations on inner states improves the children's understanding of the mind and their use of psychological lexicon.

Specifically, there is a growing body of evidence supporting a social interactionist framework in which parents input facilitates the development of children's social understanding (Ruffman et al., 2002; Taumoepeau and Ruffman, 2006, 2008; Rollo and Farris, 2012). Ruffman et al. (2002) explained the correlation between mothers' mental state utterances and children's theory of mind and “this relationship held even when many potentially mediating variables were accounted for, including the children's language ability, their initial social understanding (as manifested in their initial theory of mind and mental state language), their age and the mothers' educational background” (Rollo and Farris, 2012, p. 275).

Also, other studies found that it is a composite series of utterances that correlates with later children's theory of mind performance (psychological terms like *think, know, want, hope*). Whereas other aspects of a mothers' talk, like descriptive or causal words and links to a child's experience, seem to have a less influence on child's performances (Beeghly et al., 1986; Harris et al., 1989; Wellman and Woolley, 1990; Booth et al., 1997).

Ruffman et al. (2002), and Taumoepeau and Ruffman in two longitudinal studies (2006, 2008) showed that mothers refer most frequently to desire terms when the children are younger, whereas with older children they increase the use of belief and knowledge references. In particular, Taumoepeau and Ruffman (2006, 2008) found that maternal talk to 15-month-old children about the child's desires predicted children's mental state language and emotion task performance at 24 months. At 24 months of age mothers' reference to others' thoughts and knowledge was the most consistent predictor of children's later mental state language at 33 months.

Mothers' references to *think* and *know* increased with children's age. Thus, before 2 years of age, mother input about desire may be a mechanism by which children's emerging implicit understanding about mental life is made explicit. This mechanism can be conceptualized within the zone of proximal development (Vygotskij, 1934) such that mothers' use of specific types of mental state talk supports the child's social understanding (Rollo and Farris, 2012).

There is evidence that maternal input is linked to child mental state understanding, it has not yet been determined which maternal term is most useful for socio-emotional and cognitive development of the child, and which aspect of the cognitive development benefits the mother-child interaction.

The present study addresses this issue and focuses on the relation between mothers' mental state talk and children's performances in conceptual and semantic tasks and in a theory of mind task.

Therefore, the aim of the present research was to look more closely at how beliefs, desire and emotion usage in maternal language contributes to the prediction of children's theory of mind. In this light, the language (and the mothers' narrative in particular) is considered the driving force not only for the development of the socio-cognitive understanding, but also for the semantic development (conceptual, lexical, and metacognitive level in semantic relationships; Ebert, 2015).

### Factors related to mother-child narrative

Picture book reading poses an important context for promoting socio-cognitive understanding. For Fletcher and Reese (2005, p. 67) “within the picture book reading interaction, there are three components: an adult, a child and a book. Each component interacts with the other components to establish the social interaction.” What parent characteristics influence the quality of picture book reading interaction? Studies have examined distal factors such as socioeconomic level (SES) and culture, but also more proximal factors such as maternal sensitivity, parenting styles and parental beliefs. The effects of SES and culture have been studied through a myriad of research works (Fletcher and Reese, 2005; Vernon-Feagans et al., 2008), however, no large-scale studies have examined the effect of maternal psychological characteristics on maternal talk. Specifically, we were interested in examining whether maternal empathy could play a role in the frequency and quality of mothers' mental state utterances during a task that involved a picture book.

Empathy as “a core component of social cognition, and involves operations aimed at detecting other's mental states and predicting their future behavior” (Preti et al., 2011, p. 51) is a psychological characteristic that may influence mother's psychological lexicon. Indeed the literature suggests that among the critical aspects of maternal sensitivity may be empathy-related behaviors, e.g., to treat the child as an independent person with his thoughts, emotions and feelings (de Rosnay and Hughes, 2006). We have not found previous studies that established whether mothers' empathy predicts maternal psychological language. However, we expect that the empathic concern, involving both emotional and cognitive processes, would prompt mothers to use a larger proportion of internal state words.

Similarly, children's temperament “defined as average emotional state across a representative sample of life situations” (Mehrabian, 1996, p. 261), influences theory-of-mind development (Wellman et al., 2011) and could play an important role in a performances of theory of mind, as the psychological lexicon.

While the relation between temperament and linguistic development has already been investigated (e.g., Usai et al., 2009; Garello et al., 2012), few studies have examined the direct link between child temperament and child psychological language. Although various other factors influence children's theory of mind, the temperament as constitutionally base of the individual differences in emotional, motor, and attentional reactivity, could contribute to children's acquisition of theory of mind insights (Wellman et al., 2011).

In summary, the literature on maternal variables involved in the development of children's theory of mind, does not take into account: (a) the variables related to individual characteristics that may influence the psychological lexicon of both mothers and children; (b) general aspects about the influence of mothers' language on children's conceptual development. Specifically, further investigations are needed to estimate the effect of both mothers' empathy and children's temperament.

Therefore, there were two aims of this study. First, we examined mother's mental state lexicon, in order to describe its main characteristics in relation with the child's gender and age, and to test the relation between the mothers' frequency of mental state utterances and children's semantic and conceptual performance. Second, we examined aspects of the mother (empathy) and of the child's development (temperament) involved in psychological states understanding in relation to the child's age and gender. Specifically, (a) we expected to replicate the results of the previous study (Rollo and Longobardi, 2005) by identifying the maternal category of psychological lexicon and their variation connected with children's age and gender. Furthermore, (b) we expected to find not only an association between the mothers' frequency of mental state utterances and the frequency of the same categories of mental lexicon used by children, but also (c) an additional association between the psychological lexicon in maternal narrative and children's performance in definitional tasks. Lastly, (d) we sought to identify the relation between the mother's empathy quotient and psychological lexicon, and (e) between children's temperament and psychological lexicon.

## Method

### Participants

Fifty mother-child dyads were recruited from three preschools classes in a northern Italian city (*N* = 50) in accordance with local ethics committee approval.

The children (28 girls, 22 boys) ranged from 3 to 6 years of age (mean age = 4 years and 8 months; *SD* = 9 months) and the mothers' mean age was 35.7 (*SD* = 1.6). To assess changes caused by age, the full sample of children was split into two age groups: 3–4 years (*N* = 26) and 5–6 years (*N* = 24).

According to the parents' reports, all the children speak Italian as their main language, with 90% of the mothers reporting that Italian is spoken exclusively. The remaining families reported both parents and their child speak two languages.

Only 22% of the mothers had a foreign origin but spoke fluent Italian. The mothers indicated their completed education: 60% had completed 3–5 years of university; 30% had completed lower secondary school (*Scuola secondaria di primo grado*), which corresponds to Middle School, and only 10% had not pursued any studies after completing upper secondary school (*Scuola secondaria di secondo grado*), which corresponds to the high-school level. All mothers were of middle-class socioeconomic status (SES).

### Procedure

The criteria used for the sample selection were as follows: (a) the age of the children ranged between 3 and 6 years; (b) both mothers and children did not report any mental or physical problems or disorders and, (c) both mothers and children spoke Italian. A group of trained psychologists contacted a school and presented the research rationale and objective. Mothers who agreed to be recruited for the study filled out an informed consent form. The form was composed by two parts: the research description and quotation of the relevant law (Legislative Decree no. 196/2003, “Codice in materia di protezione dei dati personali”- Code Privacy), which was retained by the mothers; the signature sheet expressing the consent for their participation into the project together with agreement on data disclosure, which was retained by the researcher. All mothers and children met in a quiet room in the school for the following two sessions: (1) The mothers read a picture book to their children. Their shared reading was recorded and coded by two trained independent raters who listened to the audio recording and scored them on the basis of the psychological lexicon manual (described later) using a paper-pencil system. (2) After a few days, the definitional skills of children were assessed with the VCS-Assessment Test of Conceptual and Semantic Development for preschoolers (Valutazione dello sviluppo Concettuale e Semantico in età prescolare, Bellacchi et al., 2010).

In addition, the mothers were given two additional reports to be completed independently and at their home. These included a self-report measure for the assessment of their empathy status- the Empathy Quotient (EQ, Baron-Cohen and Wheelwright, 2004)—and a report-form questionnaire to evaluate their child's temperament-the Italian Questionnaires on Temperament (Questionari Italiani del Temperamento-QUIT, Axia, 2002).

## Materials

### Psychological lexicon measures

The mothers read a picture book consisting of 21 images arranged in chronological order (“Frog, where are you?,” Mayer, 1969) to their children. The maternal and children narratives were recorded, transcribed and coded by two trained independent raters. For each narrative, the proportion of mental words/total used words was calculated. Reliabilities were calculated for each code able utterance, over both child and mother utterances together. Interrater reliabilities (Cohen's k) ranged between 0.80 and 0.92.

The internal state words produced during the mother-child picture-book narratives were coded according to the categories of mental state references used in literature. The following 11 category code scheme was applied to the internal state words (Camaioni et al., 1998 and Ruffman et al., 2002 modified; Rollo and Longobardi, 2005) used by the mothers and children: (1) positive emotional words (e.g., to love, to enjoy, to be friends); (2) negative emotional words (e.g., to be afraid, to become angry); (3) cognitive words (e.g., to know, to understand, to remember, to think); (4) perceptual words (e.g., to hear, to see, to look, to observe, to recognize, to be cold, to be hot, to feel ill); (5) moral words (e.g., to forgive, to obey, to apologize, to repent, to be good, to be bad); (6) words referring to obligation (e.g., duty = to be obliged to do, power = to have the permission to do); (7) volitional words (e.g., to want, to look for, to wish); (8) ability state words (e.g., to be able, to attempt); (9) physiological words (e.g., to be hungry, to be thirsty, to be sleepy); (10) words referring to emotional displays (e.g., crying, smiling, laughing), although these utterances had strong links to emotions, they were coded separately because they described external manifestations; and, (11) communicative words (e.g., to say, to tell, to call, to ask).

During the mother-child narratives there were several categories of mental state utterances referring to the protagonist of the story as simple descriptions of a picture's contents (e.g., “The child is looking at the frog”). Therefore, we coded both the 11 categories and who they were related to: mother, child, mother-child pair or protagonists of the story.

### Semantic development measures

VCS-Assessment of Conceptual and Semantic Development for preschoolers (Valutazione dello sviluppo Concettuale e Semantico in età prescolare, Bellacchi et al., 2010) was used in order to assess the definitional skills of the children.

The test consists of the following four sub-tests that assign different measures for the changes concerning semantic representations in preschool children:

1. contextualization task: assessed knowledge concerning objects or persons typical of certain places or situations;
2. classification task: evaluated the use of taxonomic relationships to categorize objects, providing two different scores, a score of classification (conceptual component = Classification) and a score of explanation of the criteria used to classify objects (metacognitive component = Explanation);
3. words memory task: assessed the use of different types of associative or semantic relationships between terms in supporting learning and retrieval from memory of links object-word (Associative, Taxonomic, or Arbitrary relationship);
4. definitional task: assessed the use of taxonomic and linguistic relations in defining words.

Specific material—stimulus and appropriate sheet of notation were available for each task. For the contextualization task, 36 figures showing various contexts/situations were given (e.g., Bathroom, Street, Playground, Farm, Sea, Circus, etc.). For this task, children had to match each object to the context it belonged to. The classification sub-test consisted of five series of images representing five categories of objects: Animals, Fruits, Furniture, Clothes, and Vehicles. Children had to indicate which object did not fit with the others. The word memory task consisted of 36 images-cue/target-words. All words were concrete nouns and the frequency of use was medium-high in the child lexicon. The child was required to remember a word associated in the presentation to a specific image. The definitional task presented, (in random order), 12 concrete words with high frequency of use: four names (cat, hat, chair, tree), four verbs (fall, eat, play, run), and four adjectives (bad, good, great, red). The child had to explain the meaning of each word, as if they had to explain it to a foreign person. After coded responses for each of the sub-tests were obtained the scores in: Contextualization task, Classification task, Explanation task, Words memory task, Associative relationship, Taxonomic relationship, Arbitrary relationship, and Definitional task (Orsolini et al., 2010).

### Mother empathy quotient

The Empathy Quotient (EQ, Baron-Cohen and Wheelwright, 2004-Italian version edited by Liliana Ruta) was a self-report questionnaire that was developed to measure the cognitive, affective, and behavioral aspects of empathy (Preti et al., 2011).

The questionnaire contains 40 empathy questions. For each question, the responses were submitted using a four-point Likert scale: “strongly agree,” “slightly agree,” “slightly disagree,” and “strongly disagree.” Each of the questions receives a score of one point if the respondent records an empathic behavior of “slightly” and 2 points if the respondent records an empathetic behavior of “strongly,” so the scores can range from 0 to 80. Scores that ranged from 33 to 52 corresponded to a person close to the mean, 53 to 63 corresponded to a person with a empathy quotient over the mean, while higher scores (over 64) referred to a person who is very empathic. A cut off score of fewer than 32 was the most useful to differentiate adults with autism spectrum conditions from controls (Baron-Cohen, 2012).

“The original version of the EQ shows acceptable internal consistency, concurrent, and convergent validity, and good test retest reliability (Baron-Cohen and Wheelwright, 2004)” (Preti et al., 2011, p. 53). For this study, the questionnaire showed good internal consistency (Cronbach's alpha = 0.74).

### Child temperament

The Italian Questionnaires on Temperament (QUIT, Axia, 2002) were validated for an Italian sample to measure child temperament from the first month after birth to 11 years of age, with four different age groups: 1–12 months, 13–36 month, 3–6 years, and 7–11 years. The 3–6 years version of the QUIT used in this study, consisted of 60 six-point likert-type questions of children's day-to-day behaviors. For each question, mothers' answers are rated on a Likert scale from 1 (“never”) to 6 (“always”). The QUIT investigates six temperamental dimensions: social orientation (pleasure in social situations and interactions with others), inhibition to novelty (emotional reaction to novelty experience), the level of motor activity (gross motor activity, speed reaction to environment experience), positive emotionality (intensity of positive emotional reactivity and expression), negative emotionality (intensity of negative emotional reactivity and expression) and attentional capacity (ability to focus attention and to shift attention from one focus to another; Garello et al., 2012). For this study, the questionnaire for the 3–6 years age group showed good internal consistency (Cronbach's alphas ranged from 0.55 to 0.77).

## Results

### Psychological lexicon categories and their relationship to children's age and gender

The first aim of this study was to describe the main characteristics inherent in the theory of mind of maternal language (psychological lexicon) developed during a picture-reading task, and to verify how the maternal psychological state references changed with children's age and gender.

We found some significant differences in mothers' psychological lexicon (see Tables 1, 2) with respect to children's gender and age. As expected, the analyses showed that mothers used a higher proportion of Cognitive and Volitional words with older children and a higher proportion of terms referred to Obligation state with boys.

**Table 1:** **Means (SDs), *F*- and *P*-values of mothers' categories of psychological lexicon and children's age**.

**Mothers' mental state words**	**Age in years**		**Fisher *F*-Test** **(1, 48)**	***p* < 0.050**
	**3-4 (*N* = 26)**	**5-6 (*N* = 24)**		
(1) Positive Emotional	0.10 (0.06)	0.12 (0.07)	0.46	n.s.
(2) Negative Emotional	0.08 (0.05)	0.08 (0.06)	0.10	n.s.
(3) Cognitive	0.08 (0.04)	0.14 (0.11)	4.88	0.032
(4) Perceptual	0.32 (0.14)	0.26 (0.15)	2.56	n.s.
(5) Moral	0.01 (0.01)	0.01 (0.01)	0.37	n.s.
(6) Obligation	0.05 (0.05)	0.04 (0.04)	0.48	n.s.
(7) Volitional	0.07 (0.07)	0.12 (0.10)	4.27	0.044
(8) Ability	0.04 (0.04)	0.04 (0.04)	0.29	n.s.
(9) Physiological	0.06 (03)	0.13 (0.04)	0.78	n.s.
(10) Emotional Displays	0.01 (0.01)	0.03 (0.05)	2.75	n.s.
(11) Communicative	0.14 (0.07)	0.14 (0.08)	0.00	n.s.

**Table 2:** **Means (SDs), *F*-and *P*-values of mothers' categories of psychological lexicon and children's gender**.

**Mothers' mental state words**	**Gender**		**Fisher *F*-Test** **(1, 48)**	***p* < 0.050**
	**Girls (*N* = 28)**	**Boys (*N* = 22)**		
(1) Positive Emotional	0.12 (0.07)	0.10 (0.06)	0.410	n.s.
(2) Negative Emotional	0.08 (0.05)	0.07 (06)	0.571	n.s.
(3) Cognitive	0.10 (0.09)	0.12 (0.07)	0.422	n.s.
(4) Perceptual	0.31 (0.14)	0.27 (0.14)	0.888	n.s.
(5) Moral	0.01 (0.01)	0.01 (0.01)	0.016	n.s.
(6) Obligation	0.02 (0.03)	0.06 (0.06)	7.371	0.009
(7) Volitional	0.07 (0.07)	0.11 (0.10)	1.695	n.s.
(8) Ability	0.04 (0.04)	0.04 (0.03)	0.363	n.s.
(9) Physiological	0.06 (04)	0.14 (0.08)	1.240	n.s.
(10) Emotional Displays	0.02 (0.03)	0.02 (0.03)	0.006	n.s.
(11) Communicative	0.14 (0.09)	0.15 (07)	0.258	n.s.

The remaining mean scores under the two age groups were similar, although younger children scored marginally higher on Perceptual terms than older children.

The mean scores under the two gender groups were similar, although girls scored marginally higher on Positive and Negative Emotional and Perceptual terms than boys.

In addition, Analysis of Variance (ANOVA) showed a significant difference between the mothers of older children and the mothers of younger children and who they referred to while reading the story. The mothers of older children were more likely to refer to themselves, the children, or both [e.g., “have you got what's happened?”; “do you like this little dog?”; *M* = 0.03 vs. 0.01; *F*_(1, 48)_ = 8.18; *p* = 0.006]; the mothers of younger children were used to referring more frequently to the story itself [e.g., “the little dog is happy”; “the child learned that the frog had ran away”; *M* = 0.75 vs. 0.68; *F*_(1, 48)_ = 4.02; *p* = 0.05]. With respect to the characteristics of the children's language produced during the picture-reading task, although children have had little psychological lexicon, the analyses showed that the older children used, on average, a proportion higher of terms that referred to Obligation state [*M* = 0.07 vs. 0.01; *F*_(1, 48)_ = 6.186; *p* = 0.016] and that boys produced more terms that referred both to Negative emotional states [*M* = 0.06 vs. 0.01; *F*_(1, 48)_ = 4.80; *p* = 0.033] and Cognitive states [*M* = 0.20 vs. 0.06; *F*_(1, 480)_ = 6.77; *p* = 0.012]. Finally, boys had more numerous references to themselves [*M* = 0.14 vs. 0.03; *F*_(1, 48)_ = 5.67; *p* = 0.021].

### Correlations between categories of mental lexicon (mothers/children)

We also expected to find an association between the mothers' frequency of mental state utterances and the frequency of the same categories of mental lexicon produced by children. There were no significant correlations between the terms used by mothers and those produced by the children during the shared reading, however the children had a poor psychological lexicon. This is probably because the reading task was interpreted by the mothers as a task, where they had to talk, and the child had a passive role. Therefore children did not talk much. For this reason we could not investigate any relationship between mothers' words and children's words.

### Semantic development measures

In considering the scores obtained by the children in the test VCS, in all the analyses conducted, the child's gender showed no significant effect on the semantic-conceptual measures.

Regarding the child's age, a series of ANOVAs that were carried out on the scores obtained from the test VCS-Assessment of Conceptual and Semantic Development in preschool age (*Valutazione dello sviluppo Concettuale e Semantico in età prescolare*, Bellacchi et al., 2010) showed a significant difference in the scores obtained by children aged 3–4 and 5–6 years in some sub-tests (see Table 3). Older children received on average higher scores than the younger children in the Contextualization task (*p* = 0.006), and in the Definitional task (*p* = 0.001). However, there were no significant differences in the other tasks (in Classification task *p* = 0.074).

**Table 3:** **Means (SDs), *F*-and *P*-values of semantic development and children's age**.

**Semantic-Conceptual Tasks**	**Age in years**		**Fisher *F*-Test** **(1, 48)**	***p* < 0.050**
	**3-4 (*N* = 26)**	**5-6 (*N* = 24)**		
Contextualization	29 (4.62)	32 (2.39)	8.434	0.006
Classification	8.2 (2.49)	9.3 (1.74)	3.342	n.s.
Explanation	12 (9.09)	15 (6.47)	2.095	n.s.
Words Memory	20 (5.16)	21 (4.71)	1.486	n.s.
Associative	49 (35.38)	61 (34.82)	1.580	n.s.
Taxonomic	44 (24.02)	48 (24.11)	.265	n.s.
Arbitrary	39 (19.20)	39 (20.63)	0.004	n.s.
Definitional	16 (8.28)	24 (7.51)	12.931	0.001

### Psychological lexicon and semantic development

Table 4 reports the correlations between the categories of children's psychological lexicon and their semantic development. In particular, correlations between Cognitive utterances and Explanation and Associative tasks were statistically significant. In addition, both Contextualization, Words memory and Definitional Tasks correlated with Obligation state terms. These results confirm the association between different aspects of child semantic competence which are reflected both in language and in conceptualization.

**Table 4:** **Correlations between children's frequency of mental state utterances and their conceptual performances**.

**Children's mental state words**	**Children's' Semantic-Conceptual Performances**							
	**Contex**	**Class**	**Expl**	**Words**	**Assoc**	**Tax**	**Arb**	**Def**
(1) Positive Emotional	−0.03	−0.15	−0.17	−0.07	−0.17	−0.11	0.06	−0.16
(2) Negative Emotional	0.21	0.09	0.10	0.34^*^	0.06	0.20	0.23	0.28
(3) Cognitive	0.09	0.23	0.28^*^	0.28^*^	−0.04	0.09	0.26	−0.01
(4) Perceptual	−0.21	0.11	−0.04	−0.09	0.13	−0.06	0.03	−0.18
(5) Moral	0.08	0.17	0.17	−0.01	0.12	−0.08	−0.22	0.29^*^
(6) Obligation	0.30^*^	0.17	0.20	0.31^*^	0.18	0.12	−0.02	0.44^**^
(7) Volitional	0.03	0.17	0.22	0.03	0.08	−0.02	−0.20	0.32^*^
(8) Ability	−0.01	0.08	−0.02	0.19	0.11	−0.29^*^	0.20	−0.05
(9) Physiological	−0.01	0.08	−0.02	0.19	0.11	−0.29^*^	0.20	−0.05
(10) Emotional Displays	−0.25	0.10	−0.12	−0.21	0.00	−0.14	−0.09	−0.08
(11) Communicative	−0.13	0.12	0.15	0.14	0.12	0.05	−0.03	0.11

* p < 0.05;

** *p < 0.01, N = 50*.

*Legend: Contex, Contextualization task; Class, Classification task; Expl, Explanation task; Words, Words memory task; Assoc, Associative relationship; Tax, Taxonomic relationship; Arb, Arbitrary relationship; Def, Definitional task*.

By using a multiple regression we examined the relationship between mothers' mental state utterances (as Positive Emotional words, Negative Emotional words, Cognitive, Perceptual, etc.) and children's semantic and conceptual skills (as Contextualization, Classification, etc.), while controlling for both children's age in months and gender. A multiple regression was calculated to predict Contextualization score based on age, gender, and mothers' mental state utterances. A significant regression equation was found [*F*_(13, 36)_ = 2.782; *p* < 0.01] with an *R*^2^ of 0.50. Children's age was the most relevant variable in determining a change in Contextualization task (β = 0.58; *t* = 3.902; *p* < 0.001; 95% CI: 0.107–0.338); as children's age increases by one month, Contextualization score increases by 0.22.

Mothers' mental state utterances—while controlling both children's age and gender—did not significantly predict other conceptual and semantic task.

A multiple regression was calculated to predict Definitional score based on age, gender, and mothers' mental state utterances. A significant regression equation was found [*F*_(13, 36)_ = 2.532; *p* < 0.05] with an *R*^2^ of 0.49. Children's age was the most relevant variable in determining a change in Definitional task (β = 0.65; *t* = 4.227; *p* < 0.001; 95% CI: 0.281–0.799); as children's age increases by 1 month, Definitional score increases by a half point (*B* = 0.54).

By using a multiple regression we examined the relationship between mothers' mental state utterances and children's semantic and conceptual skills, while controlling for children's age in months, children's gender, mothers' empathy, and children's temperament. The model did not significantly predict children's conceptual and semantic skills, except that Contextualization. In Contextualization case, a significant regression equation was found [*F*_(20, 29)_ = 2.262; *p* < 0.5] with an *R*^2^ of 0.61. Children's age was the most relevant variable in determining a change in Contextualization performance (β = 0.56; *t* = 3.367; *p* < 0.01; 95% CI: 0.084–0.346); as children's age increases by 1 month, Contextualization increases by 0.215.

Given that we found children's performance in Contextualization and Definitional tasks to be influenced by their age, we wanted to see how maternal lexicon (dependent variable) changes depending on both children's contextualization and definitional scores at different ages. In each age group, we split both Contextualization and Definitional scores in “high scores” (≥50° percentile) and “low scores” (≥50° percentile), based on the comparison between the scores in this sample and normative scores (Bellacchi et al., 2010). As the two groups were not balanced, we ran the Mann-Whitney *U*-Test and the analysis revealed that, as regard the *Contextualization tasks*, there were no statistically significant differences in maternal lexicon when younger children (3-4 years old) had high scores (*N* = 18) or low scores (*N* = 8). Nevertheless, it seems that the mothers addressed lexical categories preferentially to their child, when they were in the “low scores” category (*p* = 0.045), or preferentially to the main character of the story, when children were in the “high scores” category (*p* = 0.045). As regard older children (5-6 years old), the ANOVA revealed a statistically significant difference between the two groups in Ability (e.g., to be able or to attempt): the mothers whose children had had high scores (*N* = 12) were used to using ability state words more [*F*_(1, 22)_ = 6881; *p* = 0.016] than the mothers whose children had had low scores (*N* = 12).

As regard the younger children's (3-4 years old) *Definitional tasks*, the Mann-Whitney *U*-Test showed statistically significant differences in maternal lexicon between children with high scores (*N* = 17), and children with low scores (*N* = 9). The mothers whose children had had high scores were more likely to use Ability state words (*p* = 0.020); the mothers whose children had had low scores were more likely to use Cognitive words (*p* = 0.007). There were no statistically significant differences in older children (5–6 years old).

### The empathy quotient

In this study, we also investigated some aspects of the mothers' empathy that may be involved in understanding of the mind. Mothers were divided into two groups depending on the scores reported: Low (*N* = 28), mothers close to the mean with scores ranging from 33 to 52, and High (*N* = 22), mothers with an empathy quotient over the mean with scores ranging from 53 to 80 (see Table 5).

**Table 5:** **Means (SDs), *F*-and *P*-values of mothers' categories of psychological lexicon and mothers' empathy quotient**.

**Mothers' mental state words**	**Empathy Quotient**		**Fisher *F*-Test** **(1, 48)**	***p* < 0.050**
	**Low (*N* = 28)**	**High (*N* = 22)**		
(1) Positive Emotional	0.10 (0.06)	0.12 (0.07)	1.80	n.s.
(2) Negative Emotional	0.07 (0.04)	0.09 (0.06)	1.23	n.s.
(3) Cognitive	0.10 (0.07)	0.12 (0.10)	0.31	n.s.
(4) Perceptual	0.30 (0.13)	0.28 (0.16)	0.18	n.s.
(5) Moral	0.01 (0.02)	0.00 (0.00)	2.94	n.s.
(6) Obligation	0.04 (0.06)	0.03 (0.03)	0.42	n.s.
(7) Volitional	0.09 (0.08)	0.09 (0.09)	0.01	n.s.
(8) Ability	0.03 (0.03)	0.05 (0.04)	4.35	0.042
(9) Physiological	0.06 (0.04)	0.14 (0.04)	0.91	n.s.
(10) Emotional Displays	0.02 (0.03)	0.02 (0.03)	0.02	n.s.
(11) Communicative	0.14 (0.08)	0.15 (0.08)	0.13	n.s.

There was a significant statistical difference between the two groups: more emphatic mothers encouraged their children more by using words that were referred to Ability states (“you're capable, you can do it”). This result was also confirmed by the significant correlation between the score of empathy (Empathy Quotient considered as a continuous measurement) and the category relating to Ability terms (*R* = 0.39; *p* = 0.005). Although the differences were not significant, mothers with high empathy quotient more frequently used Emotional words (both Positive and Negative), and also Cognitive and Physiological words.

### Children temperament

In a final step (in response to objective “e”), we expected to identify a relationship between the children's temperament and psychological lexicon. There was only one significant correlation (*R* = −0.34; *p* < 0.05) between mothers' utterances referred to Obligation states and the children temperament dimension of Social orientation - with increasing child social orientation, the obligation state in maternal language decreases and vice versa. The results also showed some significant correlations between the psychological lexicon used by children and their temperament dimensions: Attention and Positive Emotional terms (*R* = −0.29; *p* = 0.041); Positive emotionality and Perceptual terms (*R* = 0.38; *p* = 0.006); Negative emotionality and Perceptual terms (*R* = 0.39; *p* = 0.006). Therefore, children who used more emotional words had lower scores in attention, and children who used more perceptive terms had higher scores in both (positive and negative) emotional dimension of temperament.

## Discussion

The present study was supported by Vygotskij's thesis that culture and society (e.g., family) play an important role in facilitating the acquisition of higher order mental functioning. In particular, the cooperative task of shared reading enables the child to internalize ways of thinking through exposure to conversations about “thinking” with adult partners (Taumoepeau and Ruffman, 2008).

The main research question of this study was whether there was an important relation between a mother's narratives, with particular reference to the frequency and variety of psychological state terms, and the conceptual and semantic skills of children in tasks of contextualization and classification, memory and definition of words.

In studying the linguistic correlates of ToM, particular attention has been devoted to psychological lexicon or mental-state language. Taumoepeau and Ruffman (2006) have examined the relation between mother's mental state references and a later child's desire and emotion understanding. With regard to this issue, the most important result was that mothers who talk about psychological states promote their children's mental-state understanding. The effect of maternal language is not restricted to false belief understanding. It also applies to the later understanding of belief-based emotions. Why mothers' emotional utterances relate to the development of child socio-emotional understanding? Part of the explanation can be accounted for by general word learning, such that children learn about mental state terms in the same way that they learn about ordinary language (Huttenlocher et al., 1991; Peterson and Slaughter, 2003; Taumoepeau and Ruffman, 2006).

According to Ruffman et al. (2002), Harris et al. (2005), mothers' mental descriptions predicted children's correct emotion attributions even when the sample was restricted to children who had mastered the simpler false-belief task.

Other studies have suggested that the mother's explanations of emotional states in conversation predicts children's understanding of emotion (Dunn, 2002; Meins et al., 2002).

However, it has not yet been clarified which aspect of cognitive development takes advantage of mother-child interaction during picture-book reading. The current study examined the link between mothers' mental state talk and children's semantic and conceptual skills. So, we asked: whether the theory of mind of the mothers expressed in their mental-state language is useful for the semantic-conceptual development of the children; whether there is a link between maternal words, attributed to the theory of mind in explicit form, semantic and conceptual development of children.

We found results that confirmed literature data on the changes in the maternal psychological lexicon which depend on the age and gender of children, but there were also some interesting associations between maternal language and semantic development. Compared to the literature, we first identified relations between the categories of children's psychological lexicon and their semantic development. In particular, correlations between Cognitive utterances and Explanation and Associative tasks were statistically significant. In addition to these two, both Contextualization, Words memory and Definitional Tasks correlate with Obligation states terms. These results confirmed the association between different aspects of child semantic competence which are reflected both in language and in conceptualization. By using a multiple regression we examined the relationship between mothers' mental state utterances and children's semantic and conceptual skills, but the model showed no significant results and children's age was the most relevant variable in determining a change.

The analysis confirmed that children's age and gender are more influential than maternal lexicon on semantic and conceptual development. Maternal lexicon by itself cannot explain conceptual differences. When maternal lexicon finds expression in an interactive context, our personal development seems to be more relevant than interaction in semantic and conceptual development. Mothers modify their lexicon based on children's characteristics: they refers more to their child whether they perceive them as less expert, or to the main character of the story, when their child is expert. Moreover, mothers talk more about their child ability when they are more capable in contextualization as well as in definitional tasks, while they spur on their child's memories, thoughts, and considerations, when they perceive them to be more capable.

As we have been emphasizing throughout this paper, the theory of mind understanding is not an individual construction. It is a collaborative construction that continues over many years, indeed through life. The parents with their talk in narratives or in conversational settings, control children's exposure to psychological lexicon (Taumoepeau and Ruffman, 2008).

Although the main findings were the links between the maternal lexicon and semantic and conceptual skills of their children, this study aimed also to examine specific characteristics of the shared reading between mother and child, taking into account the possible influence of mothers' empathy and children's temperaments. We believed, indeed, that other less investigated variables, such as personality, were worth to be evaluated. In particular, we thought that the mothers who used Emotional and Cognitive words would have been more empathetic; moreover, their children would have had better semantic and conceptual skills. Mothers who are more empathetic toward their children's feelings, should have a rich psychological lexicon, elicitate and reinforce the use of nouns and meanings. Indeed, we found that more empathetic mothers had used more Ability terms.

Though, there was only a significant difference between the mothers with higher empathy quotient and those with lower empathy quotient, we believed that this dimension of mother's personality must be explored. The role of children's temperament was less clear instead: while it is demonstrated the role that temperament has in language development (e.g., …), its effect on psychological lexicon has not been clarified yet. Although significances were weak, ur results suggest that the effects of maternal vocabulary cannot be separated from important dimensions of personality, such as temperament and empathy.

## Limitations

This study has limitations. First, we did not control for factors of interaction, e. g. the language development of children or the perception of the task by mothers. As we presented in the results, this may mean that the mothers interpreted the task as a task were they had to talk, and the children had a passive role. For this reason children did not talk much and we could not investigate any relationship between mothers' words and children's words. Furthermore, we used two self-report forms to assess the mothers' empathy and the children's temperament.

## Conclusions and future directions

Shared reading is an important occasion for the children to engage in the pleasure of listening, to follow along by looking at aesthetically pleasing illustrations, and to develop both socio-emotional understanding and knowledge about things and people of the world.

Grazzani and colleagues, in training studies, have suggested that the usefulness of language-based intervention in educational contexts (e.g., kindergartens), actively involves children in conversation about inner states, and consequently increases their theory of mind abilities (Ornaghi and Grazzani, 2013; Grazzani and Ornaghi, 2014). These studies consisted of three phases: pre-test, training and post-test. During training, the children listened to the stories of an illustrated story book enriched with psychological terms. The children in the training group showed gains in their understanding of mental state words and in their performance on theory of mind and emotion understanding tasks.

We intend to further our research to arrange training for the mothers, increasing their use of some categories of psychological lexicon so as to better control the effect on child development. The understanding of own and others' psychological states is influenced by educational interactions, like shared reading.

This study contained elements of novelty. The most important were: (a) the consideration of individual characteristics (mothers' empathy and children's temperament), and (b) the analysis of children's conceptual and semantic development. Future research should therefore concentrate on the role of both individual and educational variables. Among the neurobiological aspects, it would be interesting to assess the child's temperament and the empathetic provisions of the caregiver.

## Author contributions

All authors listed, have made substantial, direct and intellectual contribution to the work, and approved it for publication.

### Conflict of interest statement

The authors declare that the research was conducted in the absence of any commercial or financial relationships that could be construed as a potential conflict of interest.
